# Supplementary material for: Mechano-chemical decomposition of organic friction modifiers with multiple reactive centres induces superlubricity of ta-C
Source: Nat Commun. 2019 Jan 11;10:151. doi: 10.1038/s41467-018-08042-8 (PMC6484224; doi:10.1038/s41467-018-08042-8)
Supplement: Supplementary file 1 — Supplementary Information [file 41467_2018_8042_MOESM1_ESM.pdf]

Supplementary Information

Mechano-chemical decomposition of organic friction modifiers with multiple reactive centres induces superlubricity of ta-C

Kuwahara et al.

Supplementary Note 1. EHL film thickness calculations

List of symbols

w	applied normal load (N)
P_N	applied normal pressure (Pa)
T	temperature (°C)
v	sliding velocity (ms^{-1})
α	viscosity-pressure coefficient of lubricant (Pa^{-1})
η_0	dynamic viscosity of lubricant (Pas)
τ	shear (frictional) stress (Pa)
$\dot{\gamma}$	shear rate (s^{-1})
E_A, E_B	Young's moduli of solid A and B (Pa)
v_A, v_B	Poisson's ratios of solid A and B
r_x, r_y	reduced radius of curvature parallel and perpendicular to the sliding direction (m)
r_c	Hertz contact radius (m)
A_c	Hertz contact area (m^2)
d	penetration depth of an asperity into a flat surface (m)
R	mean radius of curvature of an asperity (m)
P_{cmax}	maximum Hertz contact pressure (Pa)
E'	reduced Young's modulus (Pa) ($1/E' = 2(1 - \upsilon_A^2/E_A + 1 - \upsilon_B^2/E_B)$)
D	ratio of reduce radii of curvature (m) ($D = r_x/r_y$)
h_{centre}	central film thickness (m)
R_{RMS}	root-mean-square (RMS) surface roughness (m)
A_{mic}	surface area scanned by 3D microscopy (m^2)
$R_{RMS, mic}$	RMS surface roughness measured in the area A_{mic} (m)
$R_{RMS, c}$	RMS surface roughness estimated in the Hertz contact area A_c (m)

q wave vector (m^{-1}) ($q = 2\pi/\lambda$, where λ is the wavelength)

q_s, q_l small and large wavelength cutoff for a surface power spectral density (m^{-1})

H Hurst exponent

G Hamrock-Dowson dimensionless material parameter ($G = \alpha E'$)

U Hamrock-Dowson dimensionless speed parameter ($U = \eta_0 v / E' r_x$)

W Hamrock-Dowson dimensionless load parameter ($W = w/(E' r_x^2)$)

L Moes dimensionless material parameter ($L = G(2U)^{0.25}$)

M Moes dimensionless load parameter ($M = W/(2U)^{0.75}$)

In order to identify the actual lubrication regimes under the experimental conditions, the lubricant film thicknesses are calculated using the Nijenbanning-Venner-Moes[1,2] formula for elasto-hydrodynamic (EHL) circular contacts. The central film thickness h_{centre} is estimated by

$$h_{\mathrm{centre}} = r_x (2U)^{0.5} \left(\left(H_{\mathrm{RI}}^{3/2} + \left(H_{\mathrm{EI}}^{-4} + H_{00}^{-4} \right)^{-3/8} \right)^{2s/3} + \left(H_{\mathrm{RP}}^{-8} + H_{\mathrm{EP}}^{-8} \right)^{-s/8} \right)^{1/s}, \quad (1)$$

with

$$s = 1.5\left(1 + e^{-1.2/H_{\mathrm{EI}}/H_{\mathrm{RI}}}\right), \quad (2)$$

$$H_{00} = 1.8 D^{-1}, \quad (3)$$

$$H_{\mathrm{RI}} = 145\left(1 + 0.796 D^{14/15}\right)^{-15/7} D^{-1} M^{-2}, \quad (4)$$

$$H_{\mathrm{EI}} = 3.18\left(1 + 0.006 \ln D + 0.63 D^{4/7}\right)^{-14/15} D^{-1/15} M^{-2/15}, \quad (5)$$

$$H_{\mathrm{RP}} = 1.29(1 + 0.691 D)^{-2/3} L^{2/3}, \quad (6)$$

$$H_{\mathrm{EP}} = 1.48\left(1 + 0.006 \ln D + 0.63 D^{4/7}\right)^{-7/20} D^{-1/24} M^{-1/12} L^{3/4}. \quad (7)$$

Material and experimental parameters are summarized in Supplementary Table 1. Lubricant dynamic viscosities η_0 at 80 °C, pressure-viscosity coefficients α, and calculated EHL central film thicknesses h_{centre} are shown in Supplementary Table 2. The actual lubrication regimes are determined by evaluating

the dimensionless lambda parameter which is defined as the ratio between the central film thickness and the combined roughness of the two surfaces

$$\lambda = h_{\text{centre}} / \sqrt{R_{\text{RMS,pin}}^2 + R_{\text{RMS,disc}}^2}. \quad (8)$$

Boundary lubrication is obtained for $\lambda \lesssim 1$, mixed lubrication for $1 \lesssim \lambda \lesssim 3$ and hydrodynamic lubrication for $3 \lesssim \lambda$[3]. The surface topography after tribological testing was measured on the pin (in the middle of the wear scar) and on the disc (in the middle of one of the reversing points) with at least two measurements per sample. A 3D microscope (Leica DCM3D) with a 150x magnification confocal objective lens was used, resulting in a measured area of $A_{\text{mic}} = 84 \times 64\ \mu\text{m}^2$. First, a z-range threshold filter was used to cut-off isolated and deep pinholes that were much larger than the roughness. Next, a Gaussian filter was applied to extract the roughness. RMS surface roughness values R_{RMS} were then calculated according to ISO 25178 (Supplementary Table 1).

Under boundary lubrication, a large local contact pressure can elastically deform ta-C asperities, decreasing the surface roughness. According to the Hertz contact theory for an elastic half-space[4], the penetration depth d and the contact radius r_c can be calculated as $d = R\left(\frac{3\pi P_{\text{N}}}{4E'}\right)^{\frac{2}{3}}$ and $r_c = \sqrt{Rd}$, where R is the radius of curvature of the asperity and E' is the reduced Young's modulus of the coating. Since $P_N \ll E'$, d is much smaller than R. Thus, the elastic deformation of asperities can be neglected in the calculation of the lubrication regime.

In principle, the roughness values R_{RMS} entering Eq. (8) should be measured by scanning a surface with the size of the contact area $A_c = \pi r_c^2$ between pin and disc. In our experiments, the contact radius $r_c = R_{pin}\left(\frac{3\pi P_{\text{N}}}{4E'}\right)^{\frac{1}{3}}$ is given by $r_c = 314\ \mu\text{m}$ and therefore the size of A_c makes such a roughness measurement difficult (especially on the pin). Thus, the roughness has to be estimated from scans over smaller areas of the size A_{mic}. The validity of these estimates crucially depends on the power spectral density (PSD) of the surfaces[5]. The PSD of most technical surfaces scales as $C(q) = C(q_l)\left(\frac{q}{q_l}\right)^{-2-2H}$ for $q_l < q < q_s$. Here, H represents the Hurst exponent that usually ranges between 0.7 and 0.9[5], q is the wave vector and q_s is the small and q_l is the large wavelength cutoff with $q_l \ll q_s$. The RMS roughness is determined by the PSD via $R_{\text{RMS}}^2 = \frac{1}{2\pi}\int_{q_L} qC(q)dq$, where $q_L = \frac{\pi}{L}$ with L the length of the scanned surface area. For $q_L = q_c = \frac{\pi}{L_c} > q_l$ the simple scaling law $R_{\text{RMS},c} = R_{\text{RMS,mic}}\left(\frac{L_c}{L_{\text{mic}}}\right)^H$ holds (with $L_c = \sqrt{A_c}$ and $L_{\text{mic}} = \sqrt{A_{\text{mic}}}$) indicating that $R_{\text{RMS},c} > R_{\text{RMS,mic}}$. Consequently, using $R_{\text{RMS,mic}}$ in Eq.

(8) would yield overestimated λ values. For the surfaces considered in this article $H \approx 0.85$, $q_l \approx 1.6\ \mu\text{m}^{-1}$ and $C(q_l) = 0.004\ \mu\text{m}^4$. Therefore, $q_c = 0.0056\ \mu\text{m}^{-1}$ and $q_{mic} = \frac{\pi}{L_{mic}} = 0.043\ \mu\text{m}^{-1}$ are much smaller than q_l, i.e. $q_c \ll q_{mic} \ll q_l$. In this case, since $C(q)$ is about constant for $q < q_l$, the RMS roughness is given by

$$R_{RMS}^2 = \frac{C(q_l)}{4\pi}\left[\left(1+\frac{1}{H}\right)q_l^2 - q_L^2\right]. \quad (9)$$

Inserting $q_L = q_{mic}$ in Eq. (9) results in $R_{RMS,mic} = 42.106$ nm, while $q_L = q_c$ reveals $R_{RMS,c} = 42.113$ nm. Thus, $R_{\text{RMS},c} \approx R_{\text{RMS,mic}}$ and therefore using $R_{\text{RMS,mic}}$ in Eq. (8) is the valid choice for the surfaces considered in our experiments.

The λ values for the C_{18} lubricants are calculated using Eq. (8). All values are below one ($\lambda \leq 0.94$, see Supplementary Table 2) and therefore our reciprocating sliding tests of these lubricants were conducted under boundary lubrication. The λ value for glycerol is only slightly larger than unity ($\lambda = 1.30$) indicating a borderline situation between boundary and mixed lubrication. In order to exclude that hydrodynamic effects contribute to the superlow friction of our glycerol-lubricated systems, additional experiments with smaller reciprocation frequencies were performed ($f = 5$ and 4 Hz). This lowers the corresponding λ values to $\lambda = 0.85$ and 0.74, respectively. These values indicate that the new experiments were performed under boundary lubrication conditions. Interestingly, the steady-state friction coefficients for these smaller frequencies remain on its superlow level ($\mu = 0.008$) suggesting that already our earlier experiment with $f = 10$ Hz and $\lambda = 1.30$ was probing the boundary lubrication regime.

In order to calculate a conservative estimate of λ, we used the maximum velocity of a reciprocating cycle. However, the pin is motionless at the end of each stroke, resulting in a reduction of the film thickness. An estimate using a simple squeeze-out theory (Eq. 4 in Supplementary Ref. 6) predicts for $f = 10$ Hz a reduced $h_{\text{centre}} = 33.4$ nm at the end of the stroke resulting in $\lambda = 0.59$. Thus, even for $f = 10$ Hz near the reversing points of the strokes our λ value is smaller than the maximum estimate given in Supplementary Table 2.

Since some of our calculated λ parameters were so close to unity (and for glycerol with $f = 10$ Hz even above unity), we report here an alternative approach to determine the friction regime. Under hydrodynamic lubrication conditions the shear stress τ in the lubricant is given by $\tau = \eta\dot{\gamma}$, where η is the effective viscosity in the Hertz contact zone and $\dot{\gamma}$ the shear rate. If we estimate the shear rate using $\dot{\gamma} = \frac{v}{h_{centre}}$, the friction coefficient μ_{HD} under hydrodynamic lubrication is given by $\mu_{\text{HD}} = \frac{\tau A_c}{w} = \frac{\eta v A_c}{w h_{\text{centre}}}$. The viscosities η of the liquids at the pressure P are calculated by $\eta = \eta_0 e^{\alpha P}$. Here, the maximum Hertz

contact pressure $P_{\mathrm{cmax}} = \frac{3w}{2\pi r_c^2} = 242$ MPa is used. For all lubricants, the estimated μ_{HD} values are much smaller (0.0011−0.0015) than our experimentally recorded friction coefficients (see Supplementary Table 2). Even for oleic acid and glycerol, μ_{HD} is less than one-fifth of μ_{exp}. Since the recorded frictional forces include the contribution from the hydrodynamic component and direct asperity interactions, it is clear that boundary lubricated asperity contact friction is the dominant contribution to the frictional behaviour of ta-C observed in Fig. 1. Especially for glycerol it becomes apparent that more than 80% of the measured friction originates from boundary lubrication.

In summary, it is safe to state that the frictional and wear behaviour of our tribosystems is generally governed by boundary lubrication and not significantly affected by lubricant viscosities. Indeed, our experiments show that there is no clear correlation between the friction coefficients and viscosities. In particular, the comparison between stearic and oleic acid is the most instructive. The central EHL film thickness for stearic acid is larger than that for oleic acid, but stearic acid yields much higher friction and wear.

Supplementary Table 1: Material and experimental parameters. Young's modulus and Poisson ratio of steel were used for the Nijenbanning-Venner-Moes calculations, while the values for ta-C were considered for the Hertz estimates of asperity deformation. For the sliding velocity the maximum in the reciprocating test given by $v_{\mathrm{max}} = 2\,\pi f a$ was used, with the frequency $f = 10$ Hz and the amplitude $a = 2.5$ mm.

normal load w (N)			50
temperature T (°C)			80
maximum sliding speed v_{max} (m s^{-1})			0.157
Young's modulus E (GPa)	ta-C		430
	100Cr6		220 [Ref. 7]
Poisson ratio ν (-)	ta-C		0.17 [Ref. 8]
	100Cr6		0.30 [Ref. 9]
radius of curvature of spherical pin R_{pin} (m)			0.1
RMS roughness R_{RMS} (nm)	octadecane	pin	89.4
		disc	92.0
	stearic acid	pin	30.5
		disc	16.5
	oleic acid	pin	18.1
		disc	25.0

elaidic acid	pin	17.0
	disc	31.0
glycerol	pin	37.1
	disc	43.3

Supplementary Table 2: Lubricant dynamic viscosities at 80°C, viscosity-pressure coefficient α calculated central EHL film thicknesses h_{centre}, Tallian parameters λ as well as ratio of calculated hydrodynamic friction coefficient μ_{HD} and total experimental friction coefficient μ_{exp} . The viscosity-pressure coefficient of stearic acid had to be estimated. Since addition of stearic acid into a mixture of oleic and palmitic acid resulted in an increase of α towards the value of pure oleic acid[10], we set the $\alpha_{\text{stearic acid}} = \alpha_{\text{oleic acid}} = 7\ \text{GPa}^{-1}$ of stearic acid.

lubricant	f (Hz)	η_0 (mPas)	α (GPa^{-1})	h_{centre} (nm)	λ	$\mu_{\text{HD}}/\mu_{\text{exp}}$
octadecane	10	1.51 [Ref. 11]	10.0 [Ref. 11]	12.6	0.10	0.01
stearic acid	10	7.68 [Ref. 12]	7.0 [Ref. 10]	32.5	0.94	0.02
oleic acid	10	5.92 [Ref. 12]	7.0 [Ref. 13]	27.6	0.89	0.14
glycerol	10	31.90 [Ref. 14]	5.4 [Ref. 15]	74.4	1.30	0.19
glycerol	5	31.90 [Ref. 14]	5.4 [Ref. 15]	48.5	0.85	0.15
glycerol	4	31.90 [Ref. 14]	5.4 [Ref. 15]	42.2	0.74	0.14

Supplementary Note 2. Stereoisomerism effect

Sliding tests of ta-C/ta-C pairs show a clear reproducible trend in the friction coefficient and wear of elaidic and oleic acid (Supplementary Fig. 1): oleic acid lubrication yields a superlubricity regime ($\mu = 0.008$), whereas elaidic acid lubrication gives slightly larger friction coefficients ($\mu = 0.017$) in the ultralow friction regime. However, our QMS simulations do not provide a significant difference in the critical pressures for anchoring and cross-linking between trans- and cis-3-heptenoic acid (Supplementary Fig. 2). Since ta-C surfaces are not homogeneous, surface chemistry strongly depends on local atomic configurations and thus numerous trajectories have to be generated to obtain a significant difference as well as stable statistics. Therefore, instead of ta-C surfaces, we here use a non-reconstructed diamond (111) surface to rule out the impact of the local atomic configurations. An unsaturated fatty acid (trans- or cis-3-heptenoic acid) initially chemisorbs on one of the diamond (111) surfaces via carboxylic polar head. The lower surface is fully passivated with hydrogen atoms, while the upper surface is chosen as an

unpassivated and unreconstructed diamond (111) surface. Supplementary Figure 1 shows different structural responses of the anchored molecules to normal pressure. Trans-3-heptenoic acid keeps the straight shape in the hydrocarbon chain and becomes parallel to the surfaces gradually when the surface separation *h* is decreased. In contrast, the cis-3-heptenoic acid can bend at the C=C double bond site. The kink in the cis-3-heptenoic acid becomes more pronounced when the surface separation *h* is decreased, which leads to the exposure of the double bond to the counter surface. The same pronounced bend is observed for oleic acid (Supplementary Fig. 2). We generate five trajectories for each molecule, and the averaged critical pressures for crosslinking are 0.44±0.13 GPa and 3.93±1.36 GPa for cis- and trans-3-heptenoic acid, respectively. These results indicate that the stereoisomerism effect has a strong influence on cross-liking and shear-induced fragmentation.

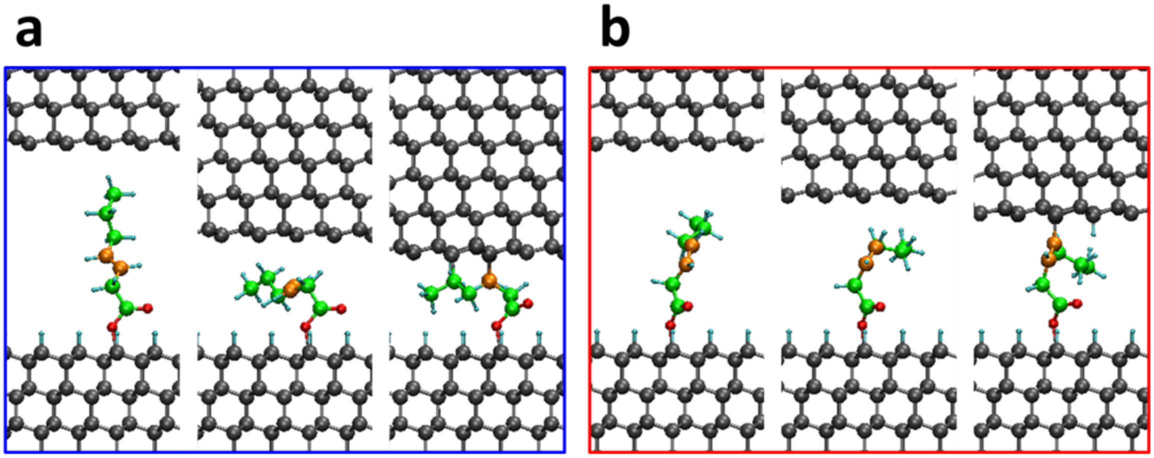

Supplementary Figure 1: Examples of QMS pressurization simulations of anchored (a) trans- and (b) cis-3-heptenoic acid.

Supplementary Note 3. QMS simulations of C_{18} fatty acids

In this study, due to the huge computational cost of quantum-mechanical simulations, we use C_7 model lubricants instead of C_{18} lubricants. However, the QM modelling with C_{18} fatty acids should be very instructive to rule out the effect of chain lengths and show the validity of our results. Therefore, we perform additional QMS simulations of C_{18} fatty acids (stearic and oleic acid) confined between two ta-C blocks. For these simulations, a larger ta-C sample is created by expanding Sample 1 (used in Supplementary Fig. 2 and 3) three times in the *x* direction. Supplementary Figure 2 shows the same ordering in the critical pressures for anchoring and cross-linking as in Supplementary Fig. 2. For oleic acid, a lower contact pressure of 2.83 GPa is required for cross-linking and it can anchor on one of the ta-

C surfaces with almost no tribological loads. A pronounced kink and the exposure of the C=C double bond to the counter surface are observed (middle panel in Supplementary Fig. 2b). Although we generate only one trajectory for each molecule, the result provides strong support for the transferability of the C_7 result to C_{18} molecules.

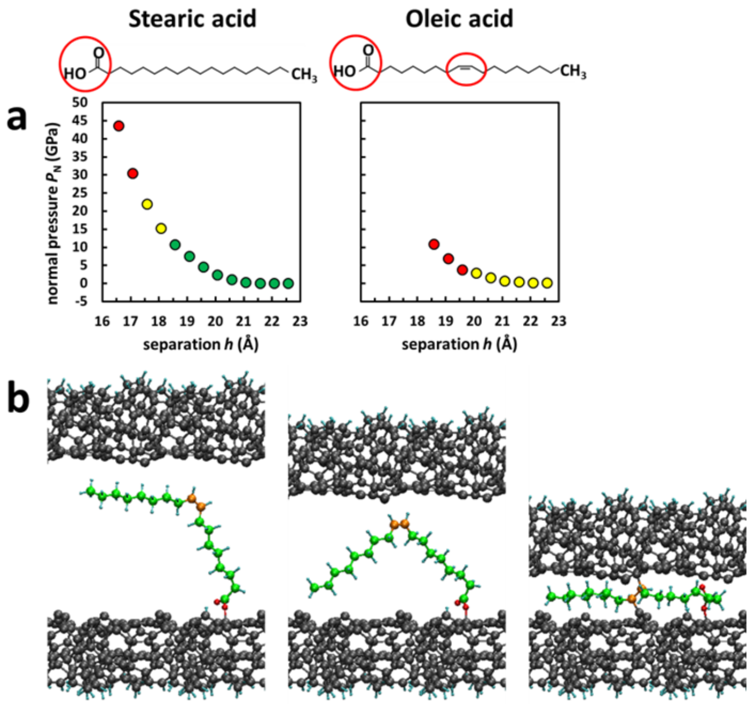

Supplementary Figure 2: Large-scale QMS simulations of C_{18} fatty acids: stearic and oleic acid. (a) Normal pressure P_N as a function of the separation h between two rigid outer C layers. (b) Snapshots for anchoring (left), bending (middle) and cross-linking (right) of an oleic acid molecule.

Supplementary References

1. Nijenbanning, G., Venner, C. H. & Moes, H. Film thickness in elastohydrodynamically lubricated elliptic contacts. *Wear* **176,** 217–229 (1994).
2. Wheeler, J., Vergne, P., Fillot, N. & Philippon, D. On the relevance of analytical film thickness EHD equations for isothermal point contacts: Qualitative or quantitative predictions? *Friction* **4,** 369–379 (2016).
3. Tallian, T. E. On Competing Failure Modes in Rolling Contact. *ASLE Trans.* **10,** 418–439 (1967).
4. Johnson, K. L. *Contact mechanics*. (Cambridge University Press, 1985).

5. Jacobs, T. D. B., Junge, T. & Pastewka, L. Quantitative characterization of surface topography using spectral analysis. *Surf. Topogr. Metrol. Prop.* **5,** 013001 (2017).

6. Persson, B. N. J. & Mugele, F. Squeeze-out and wear : fundamental principles and applications. *J. Phys. Condens. Matter* **16,** R295–R355 (2004).

7. Makowski, S., Schaller, F., Weihnacht, V., Englberger, G. & Becker, M. Tribochemical induced wear and ultra-low friction of superhard ta-C coatings. *Wear* **392–393,** 139–151 (2017).

8. Cho, S., Chasiotis, I., Friedmann, T. A. & Sullivan, J. P. Young's modulus, Poisson's ratio and failure properties of tetrahedral amorphous diamond-like carbon for MEMS devices. *J. Micromechanics Microengineering* **15,** 728–735 (2005).

9. Bradai, M. A., Braccini, M., Ati, A., Bounar, N. & Benabbas, A. Microstructure and adhesion of 100Cr6 steel coatings thermally sprayed on a 35CrMo4 steel substrate. *Surf. Coatings Technol.* **202,** 4538–4543 (2008).

10. Schaschke, C. J., Abid, S. & Heslop, M. J. High-pressure viscosity measurement of fatty acids and oils. *High Press. Res.* **27,** 33–37 (2007).

11. Ducoulombier, D. *et al.* Pressure (1-1000 bars) and Temperature (20-100 C) Dependence of the viscosity of liquid Hydrocarbons. *J. Phys. Chem.* **90,** 1692–1700 (1986).

12. Cedeno Gonzalez, P. F. O., Prieto González, M. M., Bada Gancedo, J. C. & Alonso Suárez, R. Estudio de la densidad y de la viscosidad de algunos ácidos grasos puros. *Grasas y Aceites* **50,** 359–368 (1999).

13. De Barros Bouchet, M. I. *et al.* Diamond-like carbon coating under oleic acid lubrication : Evidence for graphene oxide formation in superlow friction. *Sci. Rep.* **7,** 46394 (2017).

14. Segur, J. B. & Oderstar, H. E. Viscosity of Glycerol and Its Aqueous Solutions. *Ind. Eng. Chem.* **43,** 2117–2120 (1951).

15. Matta, C. *et al.* Superlubricity and tribochemistry of polyhydric alcohols. *Phys. Rev. B* **78,** 085436 (2008).
