# Supplementary material for: Mechano-chemical decomposition of organic friction modifiers with multiple reactive centres induces superlubricity of ta-C
Source: Nat Commun. 2019 Jan 11;10:151. doi: 10.1038/s41467-018-08042-8 (PMC6484224; doi:10.1038/s41467-018-08042-8)
Supplement: Supplementary file 3 — Description of Additional Supplementary Files [file 41467_2018_8042_MOESM3_ESM.pdf]

Description of Additional Supplementary Files

File Name: Supplementary Movie 1
Description: Quantum molecular dynamics trajectory of ta-C surfaces lubricated with a cis-3-heptenoic acid molecule.

File Name: Supplementary Movie 2
Description: Classical molecular dynamics trajectory of ta-C surfaces lubricated with octadecane molecules.

File Name: Supplementary Movie 3
Description: Classical molecular dynamics trajectory of ta-C surfaces lubricated with octadecene molecules.

File Name: Supplementary Movie 4
Description: Quantum molecular dynamics trajectory of ta-C surfaces lubricated with a glycerol molecule.
